# 

**Published:** 1875-10

**Authors:** 


					﻿[Vol. XV]
OCTOBER, 1875.
[No. 3.]
BUFFALO
Mtbicalanir jswraital lournal.
EDITED BY JULIUS F. MINER, M. D.
Professor of Special Surgery in the Euffalo Medical College ; Surgeon to the Buffalo General
Hospital; Surgeon to Buffalo Hospital of the Sisters of Charity, etc., etc.
AND
EDWARD N. BRUSH, M. D.
BTTZF’ZF' A.LO.
PUBLISHED FOR'THE EDITORS.
1875.
				

